# Public preferences for policies promoting a healthy diet: a discrete choice experiment

**DOI:** 10.1007/s10198-022-01554-7

**Published:** 2022-11-29

**Authors:** C. M. Dieteren, I. Bonfrer, W. B. F. Brouwer, J. van Exel

**Affiliations:** Erasmus School of Health Policy and Management, Erasmus University Rotterdam, P.O. Box 1738, 3000 DR Rotterdam, The Netherlands

**Keywords:** Obesity, Healthy diet, Policy, Discrete choice experiment, Public preferences, Intrusiveness, I18

## Abstract

**Background:**

Worldwide obesity rates have nearly tripled over the past five decades. So far, policies to promote a healthier diet have been less intrusive than those to reduce tobacco and alcohol consumption. Not much is known about public support for policies that aim to promote a healthy diet. In this study, a discrete choice experiment (DCE) was used to elicit stated preferences for policies varying in intrusiveness among a representative sample of the public of The Netherlands.

**Methods:**

The choice tasks presented respondents a hypothetical scenario of two policy packages, each comprising a mix of seven potential policies that differed in level of intrusiveness. We estimated mixed logit models (MXL) to estimate respondents’ preferences for these policies and performed latent class analyses to identify heterogeneity in preferences.

**Results:**

The MXL model showed that positive financial incentives like subsidies for vegetables and fruit yielded most utility. A tax of 50% on sugary drinks was associated with disutility while a tax of 20% was associated with positive utility compared to no tax at all. We identified three subgroups with distinct preferences for the seven policies to promote a healthy diet, which were characterized as being “against”, “mixed” and “pro” policies to promote a healthy diet.

**Conclusion:**

Preferences for policies promoting a healthy diet vary considerably in the Dutch population, particularly in relation to more intrusive policies. This makes selection and implementation of a policy package that has wide public support challenging.

**Supplementary Information:**

The online version contains supplementary material available at 10.1007/s10198-022-01554-7.

## Introduction

Worldwide overweight rates are alarmingly high, have nearly tripled since 1975 [1] and are expected to rise further [2]. Systematic caloric overconsumption, often referred to as an “unhealthy diet”, is the main cause of overweight and obesity in Western societies [3]. It is also one of the leading risk factors for morbidity and mortality from non-communicable diseases (NCDs) [4]. The COVID-19 pandemic further emphasized the importance of a healthy diet, as obesity is associated with more frequent hospital admissions after infection with COVID-19 [5]. Paradoxically, the imposed lockdowns to control the COVID-19 pandemic have resulted in further increases in overweight and obesity rates [6, 7].

Health policies that promote a healthy diet are expected to help reduce overweight and obesity. Designing and implementing these policies effectively is challenging since food choices—in addition to personal taste and appetite—are influenced by several exogeneous factors including price, accessibility, advertisement, social contacts, sociocultural determinants and the local food environment [8–12]. Hence, policies to promote a healthy diet require attention for a multitude of factors that potentially affect food choices in the population, therefore, requiring implementation of broader packages containing multiple targeted policies [13].

Implementing policies may also be difficult as intervening in food choices may be viewed as limiting individual’s freedom of choice and autonomy. Intrusiveness reflects the extent to which a policy intervenes in the lives of citizens [15]. One of the least intrusive health policies is the dissemination of information regarding healthy food choices (e.g., via mass media campaigns), while policies that restrict the provision of certain types of food by regulation or law are most intrusive [14, 15]. Thus far, policy initiatives promoting a healthy diet have been far less intrusive than those to reduce smoking and alcohol consumption. For the latter two, many countries have implemented taxes and age restrictions [17, 18]. Public support for tobacco control policies has increased over time, also due to awareness of the toxic character of tobacco [20].

In The Netherlands, like in most European countries, most of the implemented initiatives supporting a healthy diet aim to promote informed choice, predominantly through public information campaigns and nutrition education [21]. Only a few European countries have implemented fiscal measures such as taxes and subsidies. Denmark and Finland both have a sugar/unhealthy food tax and Denmark also decreased its taxes on sugar-free soft drinks [22]. Since 2012, France has a tax on drinks with added sugar or sweetener [23]. Taxation of unhealthy food is more common in the United States. For example, the “twinkie” tax—which increases prices of unhealthy food—has been implemented in most states [24]. Since 2010, the United States have also implemented a regulation that requires restaurant chains to display the caloric content of their servings. While this is informative and may help people in their decision-making, empirical evidence suggests that the effects on calorie consumption are relatively small [19]. One study showed that mandatory product labelling was associated with a decrease in BMI and a significantly lower probability of obesity, but this was only found among white women [25]. Regulation on food availability is most common in schools [21]. For example, in 2005, a nationwide ban on vending machines in all secondary schools was introduced in France [26]. In addition, policies to encourage healthy eating at schools, such as the free provision of fruit, have been implemented in many countries [27]. Moreover, Denmark and Switzerland regulate nutrient food content aiming to reduce trans-fatty acids [28].

While policies to promote a healthy diet in The Netherlands have been proposed, relatively few have been implemented so far. In 2018, the Dutch government and a broad coalition of parties from society and business signed the first national Prevention Agreement [29]. One of the three focus areas in this agreement was the reduction of overweight and obesity. Several goals were formulated for each focus area (e.g., reduce the overweight prevalence from 50 to 38% by 2040) and a range of policies were proposed (e.g., provision of weight loss programmes). However, the proposed policies were criticized for being insufficient to reach the stated goal of reducing overweight rates substantially [30]. More recently, the Dutch Council for Public Health and Society (RVS) published a report urging for an integrated approach, with different parties working together to reduce unhealthy lifestyles. The RVS recommended creating a legal basis for more intrusive policies aimed at stimulating healthy choices [31]. This recommendation was based on growing evidence that low intrusive policies targeting a single behaviour, such as information dissemination, have, at best, only a modest effect on behaviour [28].

Not much is known about public support for more intrusive policies promoting a healthy diet. Yet, this may be a crucial factor in designing and successfully implementing effective policies. This study uses a discrete choice experiment (DCE) to elicit stated preferences for a set of potential policies aiming to promote a healthy diet with varying levels of intrusiveness. The DCE is performed among a representative sample of the adult population in The Netherlands. Respondents were asked to choose between hypothetical scenarios of policy packages, each consisting of a mix of policies differing in level of intrusiveness. The contribution of this study is twofold. Firstly, we provide insights into public preferences for policies supporting a healthy diet in the Dutch adult population. Second, we identify and describe subgroups within the population that have different preferences regarding the proposed policies.

## Methods

We performed a discrete choice experiment (DCE) to determine preferences, among a representative sample of the Dutch adult population, for potential policies to promote a healthy diet. This DCE approach allows for an assessment of multiple attributes at the same time. Respondents were asked to choose between different policy packages, each consisting of a combination of policies differing in level of intrusiveness. The least intrusive policy was information dissemination, while the most intrusive policy was the elimination of certain food choices (see Table 1). In each choice task, respondents were asked to select the policy package that they preferred. Consequently, preferences were revealed through the respondents’ choices, also showing how respondents react to more intrusive policy measures than currently in place.

**Table 1:** Discrete choice experiment attributes and levels

Origin	Intrusiveness level	Attributes	Levels
Potentially effective policies according to literature	Eliminate choice	Ban unhealthy products from specific places	No, yes
	Restrict choice	Reduce outlets for unhealthy products	No, yes
	Guide choice through disincentives	Tax on sugary drinks	No, 20%, 50%
	Guide choice through incentives	Subsidy on vegetables and fruit	No, 10%, 30%
Proposed policies in The Netherlands	Guide choice through changing the default	Reduce serving size unhealthy products	No, yes
	Enable choice	Provide weight loss programs	No, yes
	Provide information	Show calorie content on all products/menu’s	No, yes

### Respondents

In December 2020, an online survey was distributed among a representative sample of the adult population in The Netherlands. Respondents were recruited via an independent company that hosts a large panel. Quota sampling was applied within this panel to obtain a sample of 600 respondents representative of the target population in terms of age (18–70 years), gender and level of education. To ensure representativeness of the sample, the company based the sampling quota on national statistics. Because of this relatively large sample size, we did not have to employ any parametric approach to ensure that our sample was large enough for the execution of a DCE. Ethical approval for the study was obtained from the Internal Ethical Review Board of the Erasmus School of Health Policy and Management (reference 20–06). Written consent was obtained from all respondents before the start of the survey.

### Attributes and levels

**Fig. 1:**
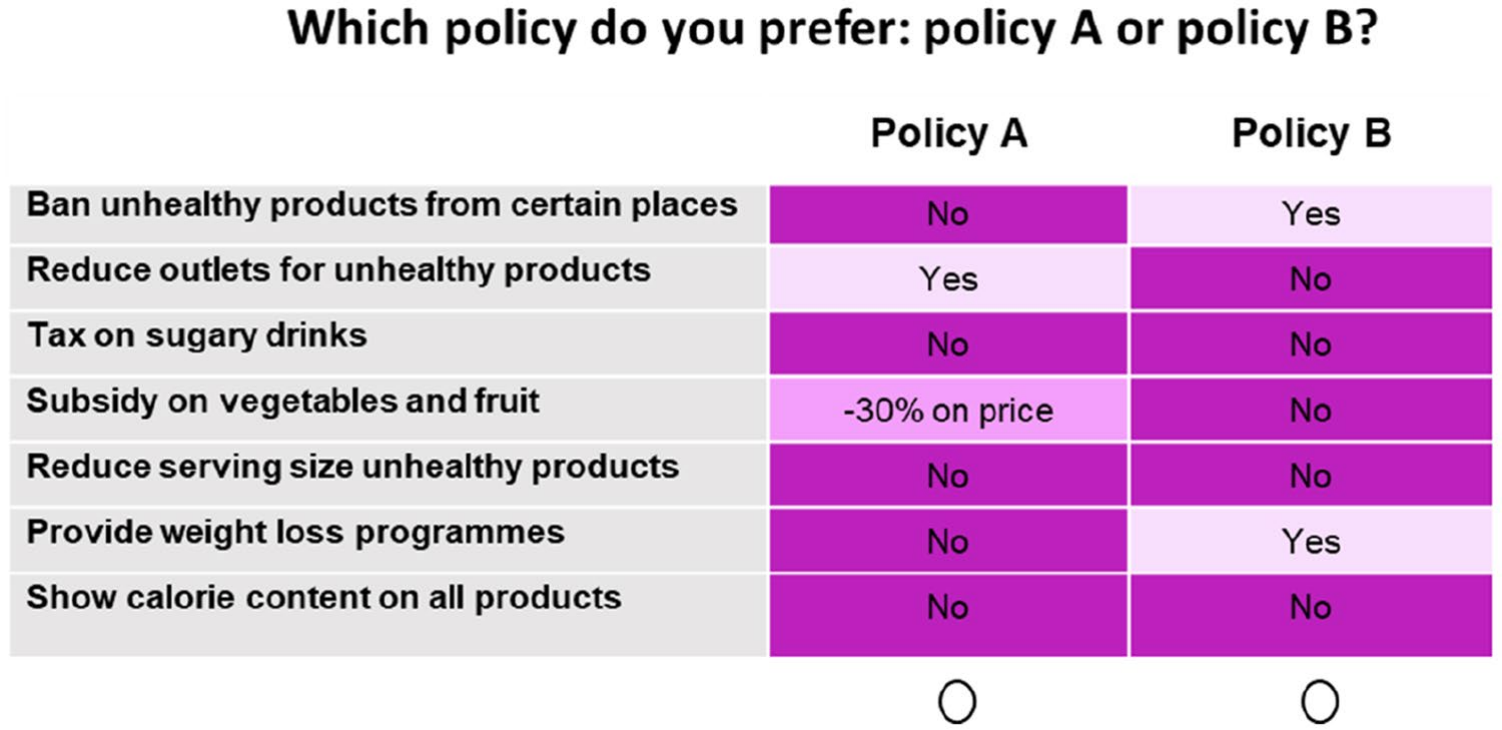
Example of a choice task presented to respondents

Respondents were asked to respond to a set of choice tasks that reflected policy packages aiming to promote a healthy diet (see Fig. 1). The Nuffield Intervention Ladder reflects how different public health policies may impact individual (freedom of) choice [15]. The ladder consists of seven “steps”, with policies higher up the ladder (top in Table 1) considered as more intrusive, i.e., more restrictive for individual choice. This ladder was used to determine the intrusiveness level of the proposed policies (see Supplementary Fig. S1). The attributes were chosen based on their relevance in current policy debates and (demonstrated or expected) positive effect on health. The choice tasks presented respondents with two policy packages consisting of a mix of the seven potential policies, indicating for each policy whether it was part of the proposed package or not. The attributes (i.e., policies) of the choice experiment and their levels (i.e., implementation) are shown in Table 1, ranked from most to least intrusive. Supplementary Table S1 includes more information on the attributes.

Including many attributes in a choice task can be burdensome for a respondent. De Bekker-Grob et al. [32] reviewed the literature and found that the vast majority of DCE studies (75%) included four to nine attributes. We included seven attributes, three of which were based on recently proposed policies in The Netherlands (i.e., the bottom three in Table 1) [29, 31]. The other four policies (i.e., the top four in Table 1) were based on policies that were suggested in the literature as having the potential of being effective [28, 33–36] and clearly are more intrusive than the proposed policies. As shown in Table 1, five of the seven attributes had dichotomous no/yes levels, which were selected to reduce the complexity and cognitive burden of the choice tasks. For the two remaining attributes (taxes and subsidies) three levels were selected based on the literature [37, 38].

### Choice tasks

The choice tasks presented to respondents consisted of the following two unlabelled policy alternatives: Policy A and Policy B. The design of the experiment included a two-step approach to account for potential disutility to respondents of all the proposed policies in a particular choice task. First, respondents were asked to choose one of the two presented policy packages by answering the question: “*Which policy do you prefer?*” (see Fig. 1) Second, respondents were asked to choose between the selected policy package or no policies implemented to promote a healthy diet by answering the question: *“You chose Policy [A/B]. If you could choose between* [the selected *Policy A/B] or no policy, what would you choose*?”.

We used colour coding to help respondents identify differences between the policies included in the two alternative policy packages without nudging respondents to focus on specific interventions [39]. Shades of purple were applied, as these have been shown to be useful in reducing cognitive burden without steering respondents in a specific direction [40]. All choice tasks had three overlapping attributes such that respondents only had to inspect the four other attributes in terms of their differences. The colour coding and the overlapping attributes were expected to improve feasibility and reduce drop-out of respondents, increasing the likelihood that respondents evaluated all policies, i.e. attribute attendance [40].

### Experimental design

A full factorial design, where respondents rate all possible combinations, would be unrealistic since this would result in 288 choice tasks (2^5^ × 3^2^: five attributes with two levels and two attributes with three levels). Therefore, a Bayesian efficient design algorithm with four attributes overlap was used to create a manageable number of 12 choice tasks [32, 41, 42]. This approach takes into account the prior parameter distributions in generation of the design [43]. The D-efficiency criterion, which leads to the minimalization of the generalized variance of the parameter estimates, was used to optimize the design [44]. To maximize the precision of the parameter estimates, heterogenous DCE designs were used [45]. This means that multiple sub-designs were simultaneously optimized. Each respondent was asked to complete only a single sub-design (consisting of 12 choice tasks) [46]. Sandor and Wedel [45] showed that compared to homogenous DCE designs, heterogenous DCE designs can be much more efficient. The different sub-designs of the survey were randomly allocated to respondents. The Bayesian design optimization algorithms were implemented with C++ programming language.

### Survey administration

Sawtooth software version 9.7.2 (Sequim, WA) was used to create the survey. Respondents received a personalized link allowing them to access the survey. The survey could be completed on any digital device. Respondents first received background information on each attribute (i.e. policy) separately (see Supplementary Table S1) to allow them to familiarize themselves with the different policies. To start with, the first three attributes were introduced and respondents were presented a trial fixed choice task consisting of these attributes. Next, the same approach was applied for the remaining four attributes. The warming-up ended with a fixed choice task consisting of all seven attributes. The attributes were presented to all respondents following the order of the Nuffield Intervention Ladder (see Table 1), that is, from lowest intrusiveness at the bottom to highest intrusiveness at the top in order to reduce the complexity of the choice tasks. A block of six choice tasks was administered, followed by and intermediate block to reduce respondent fatigue consisting of ten evaluation questions regarding the choice tasks and five unrelated questions, and concluded with a second block of six choice tasks.

### Pilot testing

We carried out a think-aloud exercise with six respondents before the start of the data collection. Respondents were asked to fill out the entire survey while thinking out loud with a researcher (CD) present. This resulted in minor changes in wording of the survey and provided an indication of how much time respondents would need to complete the survey. After this exercise, we conducted a pilot study among 100 respondents. The data from this pilot were used to optimize the priors (i.e., best guesses for the parameters) of the design, which were initially set at 0.00.

### Other variables

In between the two blocks of choice tasks, we presented respondents with seven evaluation questions about complexity and design of the choice tasks (see Supplementary Table S2). Respondents were asked to respond on a five-point Likert scale (fully disagree—fully agree). In addition, we monitored the completion time of each choice task and could thus also calculate the total completion time for the 12 choice tasks.

**Fig. 2:**
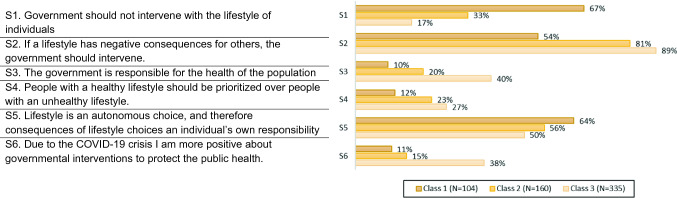
Per class the proportions that (strongly) agree with statements

Three background characteristics—age, sex and highest completed level of education—were collected at the start of the survey. In between the two blocks of choice tasks, we also asked five questions regarding household composition, employment status and financial situation. After the choice tasks, we presented the respondents with six statements regarding governmental interventions (see Fig. 2) and asked them to respond on a five-point Likert scale (fully disagree—fully agree). Statements 1, 3 and 6 were formulated specifically for this study, and statements 2, 4 and 5 were derived from previous studies [47, 48].

We also collected lifestyle characteristics and self-reported height and weight. Respondents self-reported *smoking status*, weekly alcohol consumption to identify *alcohol consumers—*consuming alcohol at least 1 day a week—and *physical activity* based on self-reported number of days with at least 30 min of physical activities per week. Based on the Dutch guidelines for physical activity, sufficient physical activity was defined as 150 min or more per week [49]. *Nutrition intake* was based on the self-reported number of days a week that respondents ate a balanced meal: appropriate portion size, not too much fat and sufficient fruit and vegetables. Sufficient variation in diet was identified as reporting to have balanced meals for at least 6 days a week [50]. We calculated *Body Mass Index *(*BMI*) with the self-reported height and weight using weight (kg)/height (m)^2^. We defined the following categories: normal weight (BMI 18.5–25.0), overweight (BMI 25.0–30.0) and obese (BMI ≥ 30.0) [51]. None of the respondents had a BMI below 18.5.

### Statistical analyses

To assess the quality of the data, we started with the examination of the evaluation questions (Supplementary Table S2) and assessed the completion time per choice task and of the entire survey. Respondents were excluded from the analyses when their average time spent on the entire survey was unrealistically short (i.e., below six minutes, this threshold was data-driven and based on the pilot results). Subsequently, we generated descriptive statistics of the background characteristics.

#### Mixed logit model

We analysed the DCE tasks under a random utility theory framework [52]. In choice task *t*, the utility *U*. of respondent *i*, associated with choosing alternative *j,* can be expressed as follows:

$$U_{ijt} = X_{ijt } b_i + \varepsilon_{ijt} ,$$

where *X* is a vector of alternative specific attribute levels, *b* represents the coefficients and *ε* the error term. The coefficients are indexed by individuals, thus acknowledging preference heterogeneity, and we assume specific distributions from the individual parameters. We estimate mixed logit (MXL) models allowing for different coefficients by respondent [53]. The random error term adjusts for individual-level variations in preferences for the corresponding attributes [54]. MXL models thus account for differences in preferences among the respondents by estimating both a mean effect and a standard deviation of effects across the sample [54]. All attributes were coded binary, with the absence of a policy as the reference category. The model estimation was conducted with 500 Halton draws with multiple starting points (random seeds) to ensure model stability [54, 55].

#### Latent class model

In addition to the MXL model, we estimated a latent class model. As we assumed heterogeneity in our sample, we wanted to assess whether this could be captured in a set of classes. Classes in this study refer to subgroups of respondents that largely share their stated preferences towards policies to promote a healthy diet. The latent class model assumes that attributes can have heterogenous effects across a predetermined number of classes [54]. This type of heterogeneity is reflected in preference weights that are identical within a class and differ systematically from preference weights estimated in the other classes [54]. The conditional logit model is used to estimate the preference weights within each class. To determine the optimal number of classes, the model diagnostics of models with 2 up to 10 classes were compared. The Akaike Information Criterion (AIC) of the models was compared to assess performance and determine the optimal number of classes, and the model with the lowest AIC was considered superior [56]. In addition, class size as well as predicted and conditional probability was compared to assess quality of the models [57]. The selected model was inspected for interpretability of the classes, and the classes were related to background characteristics of respondents and their opinion about governmental interventions. Statistical significance of the differences in these characteristics across classes was assessed using the chi^2^-test and Analysis of Variance (ANOVA).

All analyses were performed in Stata 15.0. The gllamm command was used for the latent class analyses. The mixlogit command was used to estimate the MXL models, and the mixlbeta command was used to calculate individual-level coefficients.

## Results

### Study sample description

A total of 755 respondents started with the survey and 599 completed the entire survey and spent more than 6 min on it (see Table 2). In total, 93% of the respondents indicated that the choice tasks were clear and that they considered all policy initiatives while answering the choice tasks (see Supplementary Table S2). The study sample was representative for the adult population of The Netherlands in terms of age and sex, but people with a middle level education were slightly overrepresented in the sample. Furthermore, most lifestyle characteristics were close to those of the reference population, with approximately one-fifth of the sample reporting to smoke (21%), and about half of the sample reporting sufficient variation in nutrition intake (47%), consuming alcohol on more than 1 day per week (46%), reporting insufficient physical activity levels (51%) and being overweight or obese (53%).

**Table 2:** Individual characteristics (*n* = 599)

	*n* (%)
*Demographic characteristics*	
Age, mean (SD)	48.2 (15.0)
Sex	
Female	301 (50.2)
Male	298 (49.8)
Education level^a^	
Low	122 (20.4)
Middle	291 (48.6)
High	186 (31.1)
Financial status	
Very difficult to make ends meet	23 (3.8)
Rather difficult to make ends meet	174 (29.1)
Rather easy to make ends meet	258 (43.1)
Very easy to make ends meet	144 (24.0)
Children	
Yes	329 (54.9)
No	270 (45.1)
*Lifestyle characteristics*	
Smoking	
Yes	125 (20.9)
No	474 (79.1)
Nutrition intake	
Insufficient variation	320 (53.4)
Sufficient variation	279 (46.6)
Alcohol consumption	
Yes	277 (46.2)
< 1 day p/w	322 (53.8)
Physical activity	
Insufficient	307 (51.3)
Sufficient	292 (48.8)
Weight	
Normal weight	284 (47.4)
Overweight	210 (35.1)
Obese	105 (17.5)
Body mass index, mean (SD)	26.3 (6.3)

Dutch population reference values for education: low: 26%, medium: 38%, high: 35% (www.opendata.cbs.nl)

^a^Categorization based on Statistics Netherlands (www.cbs.nl)

### Respondents’ policy preferences—MXL model

The results of the MXL model with random effects are presented in the first two columns of Table 3. All standard deviations from the coefficients were statistically different from zero indicating heterogeneity in preferences across respondents. As the reference levels of the attributes refer to an absence of the policy, positive coefficients indicate a preference for (or positive utility derived from) the corresponding policy while negative coefficients indicate a negative evaluation (or negative utility). Most utility (1.19) was derived from *30% subsidy on vegetables and fruit* (see Table 3), followed by a *10% subsidy on vegetables and fruit* (0.61). In addition, a *20% tax on sugary drinks* yielded utility, on average, while a *50% tax on sugary drinks* was associated with a disutility. Policies with lower levels of intrusiveness were generally preferred over policies with higher levels of intrusiveness, although the (differences in) coefficients were relatively small. The two most intrusive policies, *ban unhealthy products from certain places* and *reduce outlets for unhealthy products*, were not associated with significant (dis)utility, indicating that, in the overall sample, on average respondents did not significantly derive (dis)utility from these policies.

**Table 3:** Results of mixed logit regression model (MXL) and latent class analysis (LCA)

Attributes	MXL		LCA		
			Class 1	Class 2	Class 3
	Mean (SE)^a^	SD (SE)	Mean (SE)^a^	Mean (SE)^a^	Mean (SE)^a^
Ban unhealthy products from specific places	0.07 (0.05)	0.89 (0.05)**	− 0.85 (0.09)**	− 0.03 (0.06)	0.64 (0.05)**
Reduce outlets for unhealthy products	0.08 (0.04)	0.64 (0.06)**	− 0.61 (0.08)**	0.06 (0.06)	0.51 (0.05)**
50% tax on sugary drinks	− 0.55 (0.08)**	1.69 (0.09)**	− 1.92 (0.15)**	− 1.13 (0.11)**	1.04 (0.07)**
20% tax on sugary drinks	0.12 (0.05)*	0.96 (0.06)**	− 1.09 (0.10)**	− 0.40 (0.07)**	0.97 (0.05)**
30% subsidy on vegetables and fruit	1.19 (0.05)**	1.05 (0.06)**	− 0.04 (0.09)	0.98 (0.08)**	1.69 (0.06)**
10% subsidy on vegetables and fruit	0.61 (0.04)**	− 0.30 (0.07)**	− 0.18 (0.09)	0.52 (0.07)**	0.97 (0.06)**
Reduce serving size unhealthy products	0.10 (0.04)*	0.57 (0.05)**	− 0.40 (0.08)**	0.04 (0.06)	0.39 (0.05)**
Provide weight loss programs	0.33 (0.04)**	0.57 (0.08)**	− 0.20 (0.08)*	0.33 (0.06)**	0.70 (0.05)**
Show calorie content on all products/menu’s	0.16 (0.04)**	0.61 (0.06)**	− 0.23 (0.08)**	0.10 (0.06)	0.46 (0.05)**
Total *n* (%)	599 (100)		104 (17.4)	160 (26.7)	335 (55.9)
Model diagnostics					
Number of choice sets	28,752				
Log-likelihood	− 7881				
Likelihood ratio *X*^b^	1943.32				

^a^Mean *b* coefficients show estimated utility of each attribute, where positive coefficients indicate positive preference

^b^*p < 0.05; **p < 0.01

### Identification of three classes: latent class model

We found the model with three classes to have the best fit of all estimated latent class models with random coefficients. Supplementary file Table S3 shows the model diagnostics of models with two to ten classes. The jump from two to three classes showed the largest decrease in AIC, CAIC and BIC values. While these values still decrease slightly with an increased number of classes, the differences are small. Next, the models with two, three and four classes were examined regarding their interpretability and the model with three classes was considered as the most intuitive solution. In addition, the conditional probabilities (Supplementary file Table S4) showed promising values for the following three classes: the mean predicted probabilities of the allocated classes were all close to 99%, suggesting little uncertainty regarding the class that respondents were assigned to: an average score greater than 90% is considered as ideal [57]. Hence, our model performs well in distinguishing between different underlying patterns in the preferences for policy interventions to promote a healthy diet.

The the following final three columns of Table 3 show the class-specific preference estimates: Class 1 includes respondents that derive a negative utility from all policies promoting a healthy diet. Relative to the other classes, these respondents derive most disutility from negative financial incentives (*tax on sugary drinks*) and the most intrusive policies (*reduce outlets for unhealthy products* and *ban unhealthy products from certain places*). Positive financial incentives (*subsidy on vegetables and fruit*) were not significantly associated with utility in Class 1. Class 2 contains a mixture of positive and negative utility associated with policies to promote a healthy diet; negative for a *tax on sugary drinks* and positive for *subsidy on vegetables and fruit*. Class 3 contains the largest group (56%) of respondents and represents a group that derives positive utility from all proposed policies. The policies that were most preferred were the financial incentives, both as subsidy or as a tax. The least preferred policy was to *reduce serving size unhealthy products*, although its coefficient was still positive and significant.

### Latent class membership characteristics

We examined the three identified classes on demographic and lifestyle characteristics (Table 4). Class 1, the group that derived disutility from any form of policy, had the highest rates of smokers and people with overweight and obesity, but these differences across classes were not statistically significant. We only observed significant differences across classes in sex, with more females in Class 3 and fewer in Class 1.

**Table 4:** Individual characteristics by class

	Class 1	Class 2	Class 3
	*n* (%)	*n* (%)	*n* (%)
*Demographic characteristics*			
Age, mean (SD)	48.3 (12.9)	45.6 (14.6)	49.4 (15.6)
Sex			
Female	43 (41.4)	76 (47.5)	182 (54.3)^a^
Education level			
Low	27 (26.0)	32 (20.0)	63 (18.8)
Middle	52 (52.0)	76 (47.5)	163 (48.7)
High	25 (24.0)	52 (32.5)	109 (32.5)
Financial status			
Very difficult	5 (4.8)	9 (5.6)	9 (2.7)
Rather difficult	30 (28.9)	50 (31.3)	94 (28.0)
Rather easy	40 (38.5)	73 (45.6)	145 (43.3)
Very easy	29 (27.9)	28 (17.5)	87 (26.0)
Children			
Yes	55 (52.9)	84 (52.5)	190 (56.7)
*Lifestyle characteristics*			
Smoking			
Yes	29 (27.9)	33 (20.6)	63 (18.8)
Nutrition intake			
Insufficient variation	58 (55.8)	95 (59.4)	167 (49.9)
Alcohol consumption			
Yes	48 (46.1)	71 (44.4)	158 (47.2)
Physical activity			
Insufficient	48 (46.2)	81 (50.6)	178 (53.1)
Weight			
Normal weight	43 (41.4)	80 (50.0)	161 (48.1)
Pre-obesity	38 (36.5)	50 (31.3)	122 (36.4)
Obesity	23 (22.2)	30 (18.8)	42 (15.5)
BMI, mean (SD)	27.1 (7.4)	26.5 (7.4)	26.0 (5.3)
Total	17.4% (*n* = 104)	26.7% (*n* = 160)	55.9% (*n* = 335)

^a^*p*-value ≤ 0.05 between classes

Figure 2 shows the proportion of respondents per class that (strongly) agreed with the statements regarding government intervention. These findings generally coincide with the interpretations of the classes. The majority (67%) of Class 1 agreed with the statement *Government should not interfere with the lifestyle of individuals* (S1), while only 17% of respondents in Class 3 agreed with this statement. The proportion of people that believes that *the government is responsible for the health of the population* (S3) was four times larger in Class 3 as compared to Class 1. Furthermore, the responses to the statement: *Due to the COVID-19 crisis I am more positive about governmental interventions to protect the public health* (S6) in Class 3 showed a relatively positive attitude towards governmental policies to protect the public’s health, while Class 1 had a rather negative attitude to such policies (38% and 11% agreement, respectively). For all these statements, respondents in Class 2 take an intermediate position between respondents in Class 1 and Class 3. The three classes can thus be characterized as being “*against*” (Class 1), “*mixed*” (Class 2) and “*pro*” (Class 3) policies to promote a healthy diet.

## Discussion

An “unhealthy diet” is the main cause of overweight in Western societies [3] and one of the leading risk factors for morbidity and mortality from non-communicable diseases (NCDs). So far, policy initiatives to promote a healthier diet have remained less intrusive than those to reduce tobacco and alcohol consumption. We performed a discrete choice experiment (DCE) to elicit stated preferences for potential policies aiming to promote a healthy diet varying in level of intrusiveness among the public in The Netherlands.

This is one of the first DCE studies that assessed preferences for policies that support a healthy diet in relation to their level of intrusiveness. We estimated Mixed Logit (MXL) models to estimate respondents’ preferences and found, on average, that *subsidies on vegetables and fruit* yielded most utility. A moderate tax of 20% *on sugary drinks* was positively evaluated, while a higher tax of 50% was associated with disutility. This may imply that people, on average, favour a moderate tax on sugary drinks, but this should not be ‘too’ high. This finding may be explored further to see what, according to the general population, would be an optimal tax on sugary drinks.

Subsequently, we estimated a latent class model and identified three distinct classes among the respondents, which were characterized as being “against” (class 1), “mixed” (class 2) and “pro” (class 3) government intervention in individual food choices.

Previous studies mostly used a cross-sectional design including a single-item measure for public support for policies to promote a healthy diet. These studies showed that support for overweight and obesity prevention generally is high when it concerns information provision to individuals [19, 58]. These types of policies, such as mass public information campaigns, are also the most common types of action across Europe [21]. While these policies have been successful in raising awareness about unhealthy eating, their actual effect on healthy eating behaviour appears to be small [19, 28]. Public support for regulation and taxation mostly is limited [59]. Our results not only confirmed the negative evaluation of more intrusive policies in a considerable part of the respondents, but also highlighted that a small majority of our sample (56%) evaluated all the listed policies positively, including the most intrusive ones. Lanscar and colleagues [60] conducted a similar study in the Australian context, involving eight policies to reduce and prevent obesity presented together with the additional related costs and the impact on obesity rates. Interestingly, their results also revealed three classes showing a heterogeneity in policy preferences. Financial incentives to exercise were least preferred in their study [60] which contrasts somewhat with our finding that positive financial incentives were most preferred, although in our study they did not concern exercising, but subsidizing fruits and vegetables.

Public beliefs about the causes of obesity are reported to be major predictors for public support for policies to promote a healthy diet. Studies have shown that public support for policies was highest when causes for obesity were considered beyond the control of the individual (e.g. the obesogenic environment, genes) [59, 61]. Other factors, such as the lack of willpower or political view, were less relevant for policy support [58, 59, 62]. While we did not assess the beliefs about the causes of obesity, we did assess the attitude towards governmental interference with the lifestyle of individuals. This may serve as a proxy for the extent to which people believe the adoption of a healthy lifestyle is (fully) an individual’s own responsibility. We found that more than two-thirds (67%) of the “against” class believed that the government should not intervene with the lifestyle of individuals, versus only 16% of the “pro” class. This statement seems to align with a libertarian belief, emphasizing freedom of choice.

Another relevant indicator for public support is the stage of policy implementation. Currently, intrusive polices to promote a healthy diet are not common, while intrusive tobacco and alcohol policies are more prevalent. Previous research showed that public support for tobacco policies has increased over time, in particular after the introduction of smoking bans in certain areas [63, 64]. Our finding that a small majority of respondents (56%) was in favour of policies that promote a healthy diet at all levels of intrusiveness might, therefore, be explained by the fact that these policies are increasingly mentioned and are explicit topics in public and political debates [31].

It is important to note that obesity is sometimes stigmatized and, therefore, how obesity is perceived and described in policy measures, may influence the acceptance of those measures. For instance, obesity can be seen as mostly the result of environmental factors on the one hand, or as mostly due to conscious choices on the other [65]. Cawley [66] introduced an economic framework for understanding physical activity and eating behaviour and argued that individuals may fully rationally accept a higher body weight in order to gain utility derived from eating or leisure. Different perceptions regarding behavioural factors, autonomy and rationality may influence acceptability of policy measures and the type of measures considered to be necessary or acceptable if the aim is to reduce obesity. Similarly, policy actions targeted at obesity reduction may be taken in order to improve health or to improve welfare, which may not necessarily lead to the same policy choices.

### Limitations

We highlight several limitations of this study. First, data were collected during a lockdown imposed by the Dutch government to prevent the spread of COVID-19. This means that at the time of data collection respondents were confronted with invasive measures taken by the government. This extraordinary situation may have affected our results in two directions. First, some respondents may have considered the government as capable of forcefully handling the difficult public health crisis posed by COVID-19, leading to an increased recognition and trust that government intervention may improve or protect public health. On the other hand, other respondents may have disliked the imposed measures by the government and subsequently also be more prone to disfavour or distrust other measures by the government. Responses to the statement “*Due to the COVID-19 crisis I am more positive about governmental interventions to protect public health*” (S6 in Fig. 2) showed that 38% of the “pro” class agreed with this statement, while this was only 11% in the “against” class. We cannot exclude the possibility that the COVID-19 crisis had some effect on the attitude towards government interventions to protect public health, but the exact impact remains unclear. To shed more light on this, this study could be repeated in a period without such drastic measures imposed by the government.

Second, our choice tasks did not involve the potential effects on public health nor did we present the opportunity costs of the policies (e.g. who pays the subsidies for fruits). In another study [67], we explored other factors deemed relevant as a predictor for support, such as perceived effectiveness and familiarity. In the current choice tasks, we deliberately focused solely on the intrusiveness levels. Future research could further explore potential determinants for the identified preferences. Lanscar et al. [60] did include the costs and expected impact on obesity rates in a similar study and found that a large majority (78%) of their sample would support new policy and accept increased taxation. This is important since, for instance, policy measures like subsidizing vegetables and fruit would come at a price that arguably would be ultimately paid by citizens, e.g., through higher taxes. Whether or not support remains equally high in our study when confronted with the related costs is unclear. Likewise, different expectations may exist as to the (health) impact of different policy measures, which may be more or less accurate.

Third, our sample is representative of the Dutch adult population on a range of background and lifestyle characteristics. The data were collected via an online panel hosted by an independent company, and respondents received a small reimbursement for the completion of the survey. It needs noting that online data collection may lead to a certain selection of the general population, with specific segments of the population not reached via this online channel and potentially underrepresented in this study.

Finally, the DCE approach is considered as an advanced method for eliciting preferences, but a drawback is that choice tasks can be cognitively challenging for respondents. We have used a variety of methods to reduce the complexity, e.g., through colour coding and attribute overlap, but cannot exclude the possibility that a part of the respondents still experienced difficulties with evaluating the choice tasks.

## Conclusions

This study showed that preferences for policies to promote a healthy diet differ considerably in the population. More than half of the respondents (56%) in this study favoured policies to promote a healthy diet at all intrusiveness levels. This may be explained by the increased recognition that external factors like an obesogenic environment may importantly contribute to high overweight and obesity rates. Hence, governmental intervention may be considered more appropriate. However, less intrusive policies can count on the strongest public support. The implementation of more intrusive policies will likely lead to resistance. Therefore, providing additional information on these policies may be necessary. Based on our findings, a moderate sugar tax in combination with less intrusive other policies is expected to receive considerable public support in The Netherlands.

### Supplementary Information

Below is the link to the electronic supplementary material.

Supplementary file 1 (DOCX 13 KB)

## Data Availability

The data underlying this article are available in the repository of the Erasmus University Rotterdam, at 10.25397/eur.21617520.
