# Supplementary material for: Public preferences for policies promoting a healthy diet: a discrete choice experiment
Source: Eur J Health Econ. 2022 Nov 29;24(9):1429–40. doi: 10.1007/s10198-022-01554-7 (PMC9707240; doi:10.1007/s10198-022-01554-7)
Supplement: Supplementary file 1 — (DOCX 13 KB) [file 10198_2022_1554_MOESM1_ESM.docx]

**Supplementary files**

**
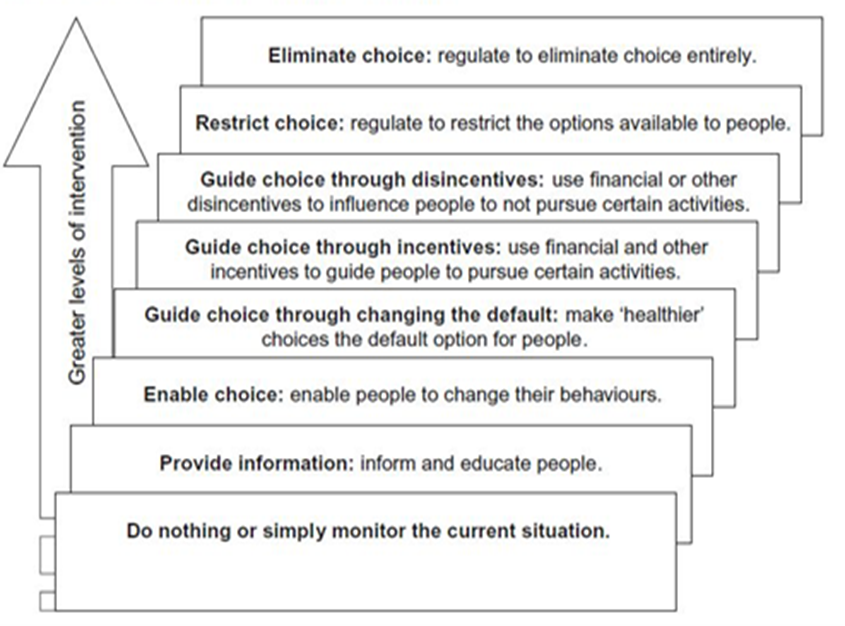
**

Figure S1. Nuffield Intervention Ladder. Source: Nuffield Council on Bioethics. Public health ethical issues. London, Nuffield Council on Bioethics, 2007.

Table S1. Background information presented with the attributes

| **Attributes** | **Background information** |
| --- | --- |
| Ban unhealthy products from certain places | At sports clubs and public transport stations unhealthy products will be removed from their range and will therefore no longer be available at these locations. |
| Reduce outlets for unhealthy products | The number of outlets for unhealthy products in your municipality will be reduced. This entails that no new outlets may be opened, or that a number of existing outlets need to be closed. |
| Tax on sugary drinks | Impose a sugar tax on all drinks that contain added sugars (e.g., soft drinks, energy drinks, sweetened water), both in supermarkets and in the catering sector. For example, a sugar tax of 20% will raise the price of a bottle of coke that costs €2,50 to €3,00. |
| Subsidy on vegetables and fruit | Give subsidy on all vegetables and fruits, both in supermarkets and in the catering sector. For example, a subsidy of 20% will lower the price of a bag of apples that costs €2,50 to €2,00. |
| Reduce serving size unhealthy products | The serving size of unhealthy products will be reduced. The price and serving size will change at the same rate. For example, a chocolate bar of 100 grams that costs 99 cents, will be reduced in size to 60 grams at a price of 59 cents. |
| Provide weight loss programmes | Each municipality offers support to people with overweight to help them loose weight by changing their behaviour. |
| Show calorie content on all products | Everywhere (for example in restaurants, cafeterias, supermarkets) the calorie content of menus or products will be shown. |

Table S2. Evaluation statements

| **Questions** | **Neutral/agree, n (%)** |
| --- | --- |
| The choice tasks were clear | 554 (92.5) |
| The choice tasks became easier after answering a few | 532 (88.9) |
| I compared all the policies before I made my choice | 567 (94.7) |
| The colours made the choice tasks easier | 513 (85.6) |
| There were too many choice tasks | 331 (55.2) |
| It was difficult to remain focused during all choice tasks | 281 (46.9) |

Table S3. Model diagnostics for 2-10 classes from latent class analysis

| **Classes** | **LLF** | **AIC** | **Δ AIC** | **CAIC** | **Δ CAIC** | **BIC** | **Δ BIC** |
| --- | --- | --- | --- | --- | --- | --- | --- |
| 2 | -7459.794 | 14957.59 |  | 15060.10 |  | 15041.10 |  |
| 3 | -7216.350 | 14490.70 | -466.89 | 14647.16 | -412.94 | 14618.16 | -422.94 |
| 4 | -7124.739 | 14327.48 | -163.22 | 14537.89 | -109.27 | 14498.89 | -119.27 |
| 5 | -7071.297 | 14240.59 | -86.89 | 14504.96 | -32.93 | 14455.96 | -42.93 |
| 6 | -7017.031 | 14152.06 | -88.53 | 14470.38 | -34.58 | 14411.38 | -44.58 |
| 7 | -6972.392 | 14082.78 | -69.28 | 14455.06 | -15.32 | 14386.06 | -25.32 |
| 8 | -6948.326 | 14054.65 | -28.13 | 14480.88 | 25.82 | 14401.88 | 15.82 |
| 9 | -6928.414 | 14034.83 | -19.82 | 14515.01 | 34.13 | 14426.01 | 24.13 |
| 10 | -6895.439 | 13988.88 | -45.95 | 14523.01 | 8 | 14424.01 | -2 |

Table S4. Conditional probabilities for 2 - 4 classes derived from latent class analyses

| **Variable** | **Obs** | **Mean** | **Std. Dev** | **Min** | **Max** |
| --- | --- | --- | --- | --- | --- |
| Max cond. prob. 2 classes | 599 | 0.9798242 | 0.0726824 | 0.5246402 | 1 |
| Max cond. prob. 3 classes | 599 | 0.9569972 | 0.1020432 | 0.5046849 | 0.999999 |
| Max cond. prob. 4 classes | 599 | 0.9570256 | 0.090794 | 0.5144748 | 0.999998 |
